# Highly Pathogenic Avian Influenza A(H5N1) Clade 2.3.4.4b Virus and Mass Mortality in Eurasian Cranes, Germany, 2025

**DOI:** 10.3201/eid3205.260170

**Published:** 2026-05

**Authors:** Anne Günther, Christof Herrmann, Julia Sehl-Ewert, Simon Piro, Ann Kathrin Ahrens, Sten Calvelage, Anne Pohlmann, Martin Beer, Timm Harder

**Affiliations:** Friedrich-Loeffler-Institut, Greifswald–Insel Riems, Germany (A. Günther, J. Sehl-Ewert, A.K. Ahrens, S. Calvelage, A. Pohlmann, M. Beer, T. Harder); Agency for Environment, Nature Conservation, and Geology Mecklenburg-Western Pomerania, Güstrow, Germany (C. Herrmann, S. Piro)

**Keywords:** highly pathogenic avian influenza, influenza, viruses, respiratory infections, zoonoses, HPAI, H5, clade 2.3.4.4b, Eurasian crane, passive surveillance, migration, transmission, Germany

## Abstract

In autumn 2025, highly pathogenic avian influenza A(H5N1) clade 2.3.4.4b virus, genotype EA-2024-DI.2.1, caused systemic infections leading to a mass mortality event among the western migrating subpopulation of Eurasian cranes (*Grus grus*) in Germany. Gregarious behavior at feeding and resting sites likely promoted rapid viral spread within the population.

Eurasian cranes (*Grus grus*) migrate along eastern, central, and western European flyways. Mass mortalities caused by goose/Guangdong (Gs/Gd)–like highly pathogenic avian influenza viruses (HPAIVs) of subtype H5 have shown susceptibility in *G. grus* cranes previously, on the eastern and central flyway in West Asia during the 2021–22 and 2024–25 HPAI seasons (*1*), and in Eastern Europe during 2023–24 (*2*,*3*). Cranes on the Western European flyway were spared from severe outbreak events, despite the ongoing HPAIV enzootic in Europe, until October 2025, when widespread deaths were detected in Germany, and thereafter in France and Spain. 

Each year, >420,000 cranes migrate through Germany (*4*). Birds migrating from Scandinavia typically roost along the coast in Mecklenburg-Western Pomerania, whereas cranes from Finland, Poland, and the Baltic region prefer inland staging sites such as Lake Galenbeck, the Müritz region, Rhin-Havelluch, or the Berga/Kelbra reservoir (Figure 1). Birds that roost in the roosting region Diepholzer fen (Figure 1) in northwestern Germany continue on to wintering areas in France and Spain.

**Figure 1:**
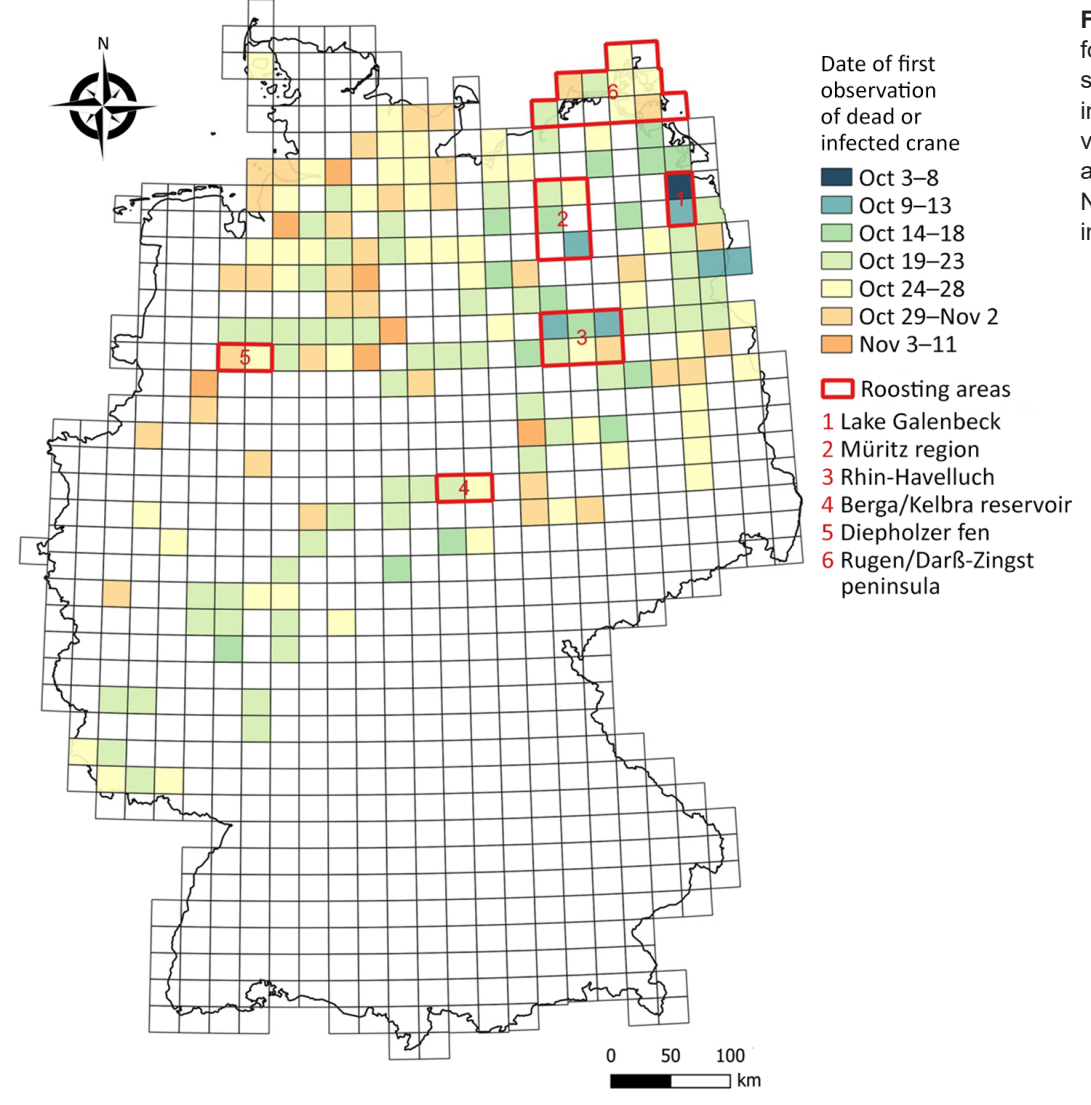
Locations of notifications for deceased or infected cranes in study of highly pathogenic avian influenza A(H5N1) clade 2.3.4.4b virus, Germany, 2025. Notifications are from the period October 3–November 11, 2025. Numbers indicate mentioned roosting areas.

The first deceased cranes in Germany were found in early October 2025 at Lake Galenbeck and were confirmed HPAIV A(H5N1)–positive shortly afterward. As of March 2026, no increased mortality has been reported from regions north (Sweden) and east (Poland and the Baltic countries) of the index site. Six cranes associated with the initial outbreak underwent necropsy. Carcasses were well preserved and in good nutritional and body condition. Gross lesions were consistent with an acute systemic process and dominated by pancreatic necrosis, pulmonary edema, and occasional epicardial and proventricular hemorrhages (Appendix 1). We examined 3 birds histopathologically. Immunohistochemistry revealed the highest influenza A virus–specific antigen loads in the central nervous system (CNS) and pancreas; moderate loads in the heart, spleen, and kidneys; and low antigen loads in respiratory and intestinal tissues. Despite widespread viral antigen distribution, necrosis was present only in subsets of antigen-positive areas. The lesion pattern largely corresponds to previous reports in cranes, particularly severe pancreatic necrosis and CNS involvement (*3*). Because immunohistochemistry was not performed in the previous study, we cannot directly compare viral antigen loads. In our cases, widespread antigen detection with only limited inflammation and necrosis suggests a peracute and rapidly progressive disease course (Figure 2; Appendix 1). Reported neurologic signs in the field, including uncoordinated movements and lack of escape behavior, correlate with widespread CNS infection.

**Figure 2:**
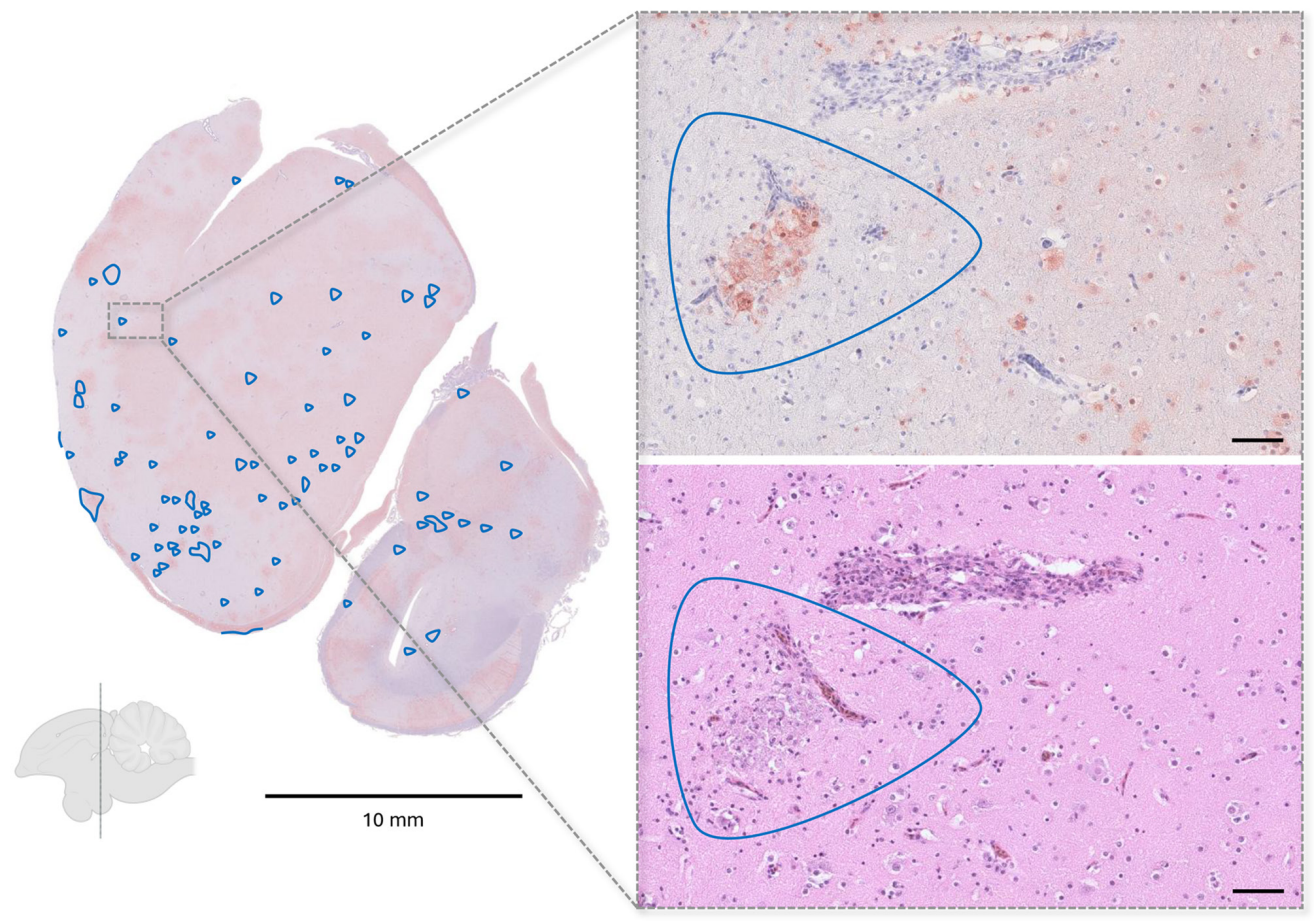
Representative histopathologic findings in influenza A(H5N1)–infected cranes from study of highly pathogenic avian influenza A(H5N1) clade 2.3.4.4b virus causing mass mortality in cranes, Germany, 2025. Immunohistochemistry of a coronal brain section at the level of the nidopallium demonstrating widespread viral antigen detection (brown staining) with only limited inflammatory and necrotic changes (blue outlines). Callout images at right show the corresponding region in consecutive sections, with abundant antigen-positive cells in immunohistochemistry (top) but only a small necrotic focus in the matching hematoxylin and eosin–stained section (bottom). Scale bars in callout images represent 50 μm.

We confirmed HPAIV H5N1 infection by real-time quantitative reverse transcription PCR in oropharyngeal and cloacal swab samples and in lung and brain tissues, finding very high virus loads (Appendix 1). Genome sequencing of 9 cranes and 25 HPAIV H5N1–positive samples from wild birds, as well as 43 samples from domestic holdings, identified HPAIV H5N1 clade 2.3.4.4b of genotype EA-2024-DI.2.1 (Figure 3; Appendix 1). Since September 2025, DI.2.1-like viruses have been detected almost simultaneously at multiple sites across Europe (*5*), appearing to be a sublineage of the Gs/Gd-like HPAIV genotype EA-2024-DI.2, which dominated the European HPAIV epizootiology in 2024 and the first half of 2025 (*6*). Phylogenetic analyses indicated that all sequences derived from HPAIV H5–positive cranes fall into a monophyletic cluster of EA-2024-DI.2.1 (Figure 3). The cluster is interspersed with sequences from domestic poultry and other wild bird species from different families. That clustering pattern in the phylogenetic tree could indicate a closely linked and direct spread within crane flocks in Germany after a primary introduction. However, our early data do not enable us to reliably distinguish direct spread from the possibilities of a diffuse dissemination in wild or domestic birds, or both, with repeated spillover to crane populations, or dissemination from an unsampled host compartment with repeated spillover to the sampled species. The detection of mild or even asymptomatic cases (e.g., in dabbling ducks) and related sequence data will help to better discern dissemination patterns in avian wildlife.

**Figure 3:**
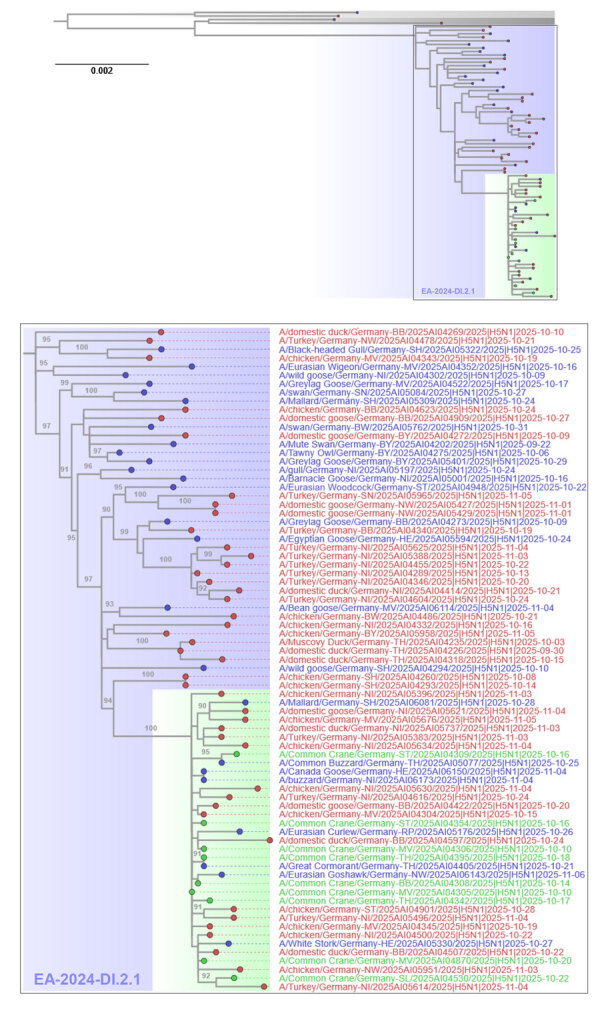
Maximum-likelihood tree based on whole-genome analyses from study of highly pathogenic avian influenza A(H5N1) clade 2.3.4.4b virus causing mass mortality in cranes, Germany, 2025. Tree at top depicts the H5N1 genotype EA-2024-DI.2.1 cluster (blue shading and text) comprising poultry (red tip) and wild bird cases (blue tip) from Germany. For comparison, recent exemplary sequences for previous dominating genotypes DI.2 and DI.1 appear (gray) above the tree. Scale bar indicates substitutions per site. In the expanded tree at bottom, gray numbers at the branches indicate bootstrap values >90%. Sequences from infected cranes (green tip) and their cluster (green shading) are highlighted. A list of virus sequences with relevant metadata is provided (Appendix 2).

Veterinary authorities and bird conservation organizations initiated carcass removal; efforts varied and were often hindered by the challenging wetland terrain. Removing carcasses has been deemed useful for reducing the incidence of infections in other gregarious species (*7*). Despite mitigation attempts, 18,164 deceased cranes were reported in Germany during October–December 2025, vastly outnumbering previously reported detections of HPAIV H5 clade 2.3.4.4b in cranes in Germany in 2020 (H5N8, n = 2) and 2023 (H5N1, n = 9).

By early November 2025, crane mortality slowed and finally almost stopped in Germany. As the cranes continued their migration, the virus spread to France and Spain. At Lake du Der-Chantecoq in northeastern France, 4,000–5,000 dead cranes were recorded during October through mid-November; an estimated 15,000–20,000 cranes died across France. In Spain, the estimated range of fatalities was 1,000–1,500, including 900 around the Gallocanta lagoon. Altogether, the estimated mortality rate along the Western European flyway in autumn 2025 was estimated at ≈10% of the crane flyway population, which comprised >420,000 cranes (*4*). The nearly identical, phylogenetically very closely related sequences of HPAIV H5N1 from Eurasian cranes suggest that, once DI.2.1 entered the population, cranes became both severely affected hosts and efficient amplifiers, contributing to rapid intraspecies-specific spread along the flyway. Close contact at night roosts in shallow waters likely accelerated the transmission of the virus through contaminated surface water; even minimal environmental viral loads in surface water have been shown to be sufficient for infecting susceptible species (*8*).

The source species and location of the current genotype DI.2.1 of HPAIV H5N1 have not been elucidated. Additional sequence data from a broader European perspective will help to identify HPAIV H5N1 outbreaks in poultry farms as possible sources or sinks of the virus and to trace transmission chains. Although cranes dominated in the clinical manifestation of the current H5N1 epizootic, they may not necessarily be the main drivers in virus spread. The same virus variant was detected in Germany simultaneously in several anseriform species, representing other possible reservoir host groups, such as geese, wigeons, and mallards (Figure 3; Appendix 1). However, when compared with that of previous seasons, mortality in those species was low and sporadic; possible causes were partial population immunity or characteristics of the current H5N1 genotype. Although those species might have been clinically less vulnerable, they were not necessarily protected from virus infection and excretion. In contrast, the crane population appeared to be fully susceptible, likely because of a lack of prior exposure to HPAIV H5 in Western Flyway populations; other unresolved intrinsic (e.g., cell receptor distributions, immune genetics) and extrinsic factors might have been involved. Unfortunately, no stored serum samples are available to confirm the assumption of a seronegative population. Furthermore, it remains unclear if HPAIV H5 circulation influenced cranes’ migratory behavior, such as avoiding heavily affected roosting sites.

As of November 17, 2025, more than 18,000 dead cranes had been reported in Germany alone in this outbreak. The full effect of the HPAI-associated mass mortality on the population structure of the Western Flyway crane population must be studied. The event highlights critical knowledge gaps in our understanding the epidemiology of HPAIV H5 in wild birds; those gaps primarily result from inadequate active monitoring and the lack of serologic data needed to assess population immunity. In addition, the networks driving HPAIV H5 transmission and spread within and between wild bird and poultry populations remain poorly understood. The characteristics of the current EA-2024-DI.2.1 genotype require detailed investigation, particularly in wild Anseriformes birds. 

Appendix 1 Additional information from study of highly pathogenic avian influenza A(H5N1) clade 2.3.4.4b virus causing mass mortality in Eurasian cranes, Germany, 2025.

Appendix 2 Virus sequence data from study of highly pathogenic avian influenza A(H5N1) clade 2.3.4.4b virus causing mass mortality in Eurasian cranes, Germany, 2025.
