# Correction: Vol. 32, No. 5

**DOI:** 10.3201/eid3206.C13206

**Published:** 2026-06

**Keywords:** errata, erratum, correction

A description of the mortality rate among cranes was unclear in Highly Pathogenic Avian Influenza A(H5N1) Clade 2.3.4.4b Virus and Mass Mortality in Eurasian Cranes, Germany, 2025 (A. Günther). The article has been corrected online (https://wwwnc.cdc.gov/eid/article/32/5/26-0170_article).

