# Supplementary material for: Highly Pathogenic Avian Influenza A(H5N1) Clade 2.3.4.4b Virus and Mass Mortality in Eurasian Cranes, Germany, 2025
Source: Emerg Infect Dis. 2026 May;32(5):779–83. doi: 10.3201/eid3205.260170 (PMC13175094; doi:10.3201/eid3205.260170)
Supplement: Appendix 1 — Additional information from study of highly pathogenic avian influenza A(H5N1) clade 2.3.4.4b virus causing mass mortality in Eurasian cranes, Germany, 2025. [file 26-0170-Techapp-s1.pdf]

Article DOI: https://doi.org/10.3201/eid3205.260170

Highly Pathogenic Avian Influenza A(H5N1) Clade 2.3.4.4b Virus and Mass Mortality in Eurasian Cranes, Germany, 2025

Appendix 1

Results

Additional Data on Migration Patterns and Mortality in Eurasian Crane (*Grus grus*) Flocks in Germany

In Germany, large concentrations of Eurasian cranes (*Grus grus*) typically occur at the Pomeranian coast in the first half of October, at the Rhin-Havelluch in the second half of October, and at the Diepholzer fen from the end of October onwards. In 2025, high-pressure weather patterns and northeastern winds caused three large migratory waves (13 and 18/19 October and 6 November), leading to increased crane densities in southern resting areas (Figure 1).

The temporal and regional distribution of fatalities associated with HPAIV H5N1 mainly followed known migration trajectories. In October 2025, an unusual backward migration toward the northeast was documented via satellite tracked and ringed birds, resulting in virus introduction into major stopover sites around Rügen/Darß-Zingst peninsula (Figure 1, #6), previously unaffected. The first carcass in this region was detected on October 19th, 16 days after the index records at Lake Galenbeck (Figure 1, #1).

The total numbers of dead cranes recorded so far are: Mecklenburg-Western Pomerania (especially Lake Galenbeck, Müritz region, Rügen/Daß-Zingst peninsula): 560; Brandenburg (especially Rhin-Havelluch, including the roost site Linum): 4,500, Saxony-Anhalt and Thuringia (especially Berga/Kelbra) ca. 6,000 and 711, respectively, Lower Saxony 6,000 (especially Diepholzer fen) (Figure 1).

Pathologic findings and histopathological analysis results are documented (Appendix 1 Tables 1, 2; Appendix 1 Figure 1). Molecular screening results are documented (Appendix 1 Table 3).

Phylogenetic and Phylogeographic Analyses

For a more detailed analysis, a subset of HPAIV sequences obtained from cranes was selected and supplemented with additional crane-derived HPAIV sequences retrieved from publicly available databases. Phylogenetic and spatiotemporal reconstruction suggests an overall gradual southward spread of the virus between October 10 and October 22 (Appendix 1 Figure 2). This pattern indicates a potential epidemiologic connection between crane roosting sites located in different regions of Germany and may reflect virus dissemination facilitated by movements of cranes between these areas.

Material and Methods

Summary of Report on Cranes with Clinical Signs and Mortality in Eurasian Cranes

Reports of deceased or infected cranes between 2025–10–01 and 2015–11–11 (n = 1398) were selected from database at https://www.ornitho.de/ and provided by the Dachverband Deutscher Avifaunisten (DDA). The TK50 grid provided by Bundesamt für Kartographie and Geodäsie (https://www.bkg.bund.de/) was used to demonstrate the timeline of the virus spread in cranes by color-coding each grid cell according to the first report of deceased or infected cranes.

Necropsy and Tissue Sampling

Six common cranes underwent necropsy. CNS, heart, lungs, pancreas, liver, spleen, kidneys, proventriculus, gizzard, small intestine, colon, and ceca were sampled and fixed in 10% neutral-buffered formalin.

Histopathology

Formalin-fixed tissues were paraffin-embedded, sectioned at 2–3 µm, and stained with hematoxylin and eosin. Necrosis and inflammation were scored on a 0–4 scale: 0 = no lesion; 1 = minimal (<5%), 2 = mild (6%–40%), 3 = moderate (41%–80%); 4 = severe (>80%).

Immunohistochemistry

IHC was performed using a mouse monoclonal antibody against the influenza A virus matrix (M1) protein (ATCC clone HB-64) (*1*). Antigen distribution was scored 0–4: 0 = no

antigen; 1 = minimal (<5%, focal-oligofocal); 2 = mild (6%–40%, multifocal); 3 = moderate (41%–80%, coalescing); 4 = severe (>80%, diffuse).

Molecular Screenings

Oropharyngeal and cloacal swabs, as well as tissue samples from brain and lungs, had been screened in RT-qPCRs targeting different genome sections: matrix protein (M1.4), hemagglutinin HA5 (H5), neuraminidase NA1 (N1) and the multibasic cleavage site of clade 2.3.4.4b HPAIV H5 (*2*,*3*).

Phylogenetic and Phylogeographic Analyses

Sequencing of avian influenza-positive samples collected in Germany 2023 was performed using an amplicon-based protocol on nanopore platforms (Oxford Nanopore Technology, Oxford, UK). Briefly, RNA was transcribed into DNA using the Superscript III One-Step and Platinum Taq kit (Thermo Fisher Scientific, USA) with Influenza A specific primers, each binding to the conserved 3′ or 5′ end of all Influenza RNA segments (Pan-IVA-1F_BsmF: TATTCGTCTCAGGG-AGCRAAAGCAGG; Pan-IVA-1R_BsmR: ATATCGTCTCGTATT-AGTAGAAACAAGG). DNA amplicons were purified with Agencourt AMPure XP magnetic beads (Beckmann Coulter, Krefeld, Germany) using DNA LoBind Tubes (Eppendorf, Wesseling-Berzdorf, Germany). Quantification of nucleic acids was done with Qubit Fluorometry (Thermo Fisher Scientific, USA). Approximately 200 ng of cDNA was sequenced using a transposase-based library preparation approach with Rapid Barcoding (SQK-RBK114, Oxford Nanopore Technologies, Oxford, UK) and on a PromethION P2 Solo instrument with the latest MinKNOW software core (v6.5.14). High accuracy base calling of the raw data using Dorado (v7.9.8, Oxford Nanopore Technologies) was followed by demultiplexing, a quality check and a trimming step to remove poor quality, adaptor and short (<20 bp) sequences. The generated data were stored in FASTQ and POD5 data formats. The bioinformatics software suite Geneious Prime (GraphPad Software LLC, version 2025.1.3) was used for analysis. Sequences were trimmed to remove primer sequences. Consensus sequences were obtained using an iterative map-to-reference approach with Minimap2 (vs 2.24) (*4*). Reference genomes were selected from a curated collection of all HA and NA subtypes and a selection of internal gene sequences to cover all potentially circulating viral strains. Polishing of the final genome sequences and annotation was performed manually after consensus generation (threshold matching 60% of bases of total adjusted quality.

Generated sequence data have been deposited in Genbank and are publicly available under accession PX662097 - PX662736.Additional sequences used in this analysis were obtained from the EpiFlu database and are available under DOI: 10.55876/gis8.251121ev. We acknowledge the originating and submitting laboratories for providing the sequence data. Segment-specific and concatenated whole-genome multiple alignments were generated with MAFFT (v7.450) (*5*) and subsequent maximum likelihood (ML) trees were calculated with RAxML (v8.2.1) (*6*) using a GTR GAMMA with rapid bootstrapping and search for the best scoring ML tree, supported by 1000 bootstrap replicates, or for large alignments with FastTree (v2.1.11) (*7*).

Time-scaled trees of concatenated genomes of the same genotype were calculated with the BEAST X (v10.5.0) (*8*) software package using a GTR GAMMA substitution, an uncorrelated relaxed clock with a lognormal distribution and coalescent constant population tree models. Phylogeographic continuous trait spatial diffusion models were calculated for genotype-based sets using a Bayesian coalescent model with latitude and longitude of the sampling. Chain lengths were set to suitable iterations and convergence checked via Tracer (v1.7.1) (*9*). Time-scaled summary maximum clade credibility trees (MCC) with 10% post- burn- in posterior were created using TreeAnnotator (v1.10.4) and visualized with FigTree (V1.4.4). The robustness of the MCC trees was evaluated using 95% highest posterior density confidence intervals at each node and posterior confidence values as branch support. The spatio-temporal diffusion models were analyzed and visualized using Spread (v.1.0.7) (*10*) and QGIS (v.3.16, QGIS.org). Geographic geojson vector maps were created with open data provided by the Federal Agency for Cartography and Geodesy (http://opendatalab.de/projects/geojson-utilities/) (https://gdz.bkg.bund.de/).

References

1. Hohensee L, Scheibner D, Schäfer A, Shelton H, Mettenleiter TC, Breithaupt A, et al. The role of PB1-F2 in adaptation of high pathogenicity avian influenza virus H7N7 in chickens. Vet Res. 2024;55:5. PubMed https://doi.org/10.1186/s13567-023-01257-8

2. Hassan KE, Ahrens AK, Ali A, El-Kady MF, Hafez HM, Mettenleiter TC, et al. Improved subtyping of avian influenza viruses using an RT-qPCR-based low density array: ‘Riems Influenza A Typing Array’, version 2 (RITA-2). Viruses. 2022;14:415. PubMed https://doi.org/10.3390/v14020415

3. Iervolino M, Günther A, Begeman L, Aguado B, Bestebroer TM, Bellido-Martin B, et al. The expanding avian influenza panzootic: skua die-off in Antarctica. bioRxiv, 2025. https://doi.org/10.1101/2025.04.25.650384.

4. Li H. Minimap2: pairwise alignment for nucleotide sequences. Bioinformatics. 2018;34:3094–100. PubMed https://doi.org/10.1093/bioinformatics/bty191

5. Katoh K, Standley DM. MAFFT multiple sequence alignment software version 7: improvements in performance and usability. Mol Biol Evol. 2013;30:772–80. PubMed https://doi.org/10.1093/molbev/mst010

6. Stamatakis A. RAxML version 8: a tool for phylogenetic analysis and post-analysis of large phylogenies. Bioinformatics. 2014;30:1312–3. PubMed https://doi.org/10.1093/bioinformatics/btu033

7. Price MN, Dehal PS, Arkin AP. FastTree 2—approximately maximum-likelihood trees for large alignments. PLoS One. 2010;5:e9490. PubMed https://doi.org/10.1371/journal.pone.0009490

8. Baele G, Ji X, Hassler GW, McCrone JT, Shao Y, Zhang Z, et al. BEAST X for Bayesian phylogenetic, phylogeographic and phylodynamic inference. Nat Methods. 2025;22:1653–6. PubMed https://doi.org/10.1038/s41592-025-02751-x

9. Rambaut A, Drummond AJ, Xie D, Baele G, Suchard MA. Posterior summarization in Bayesian phylogenetics using Tracer 1.7. Syst Biol. 2018;67:901–4. PubMed https://doi.org/10.1093/sysbio/syy032

10. Bielejec F, Rambaut A, Suchard MA, Lemey P. SPREAD: spatial phylogenetic reconstruction of evolutionary dynamics. Bioinformatics. 2011;27:2910–2. PubMed https://doi.org/10.1093/bioinformatics/btr481

Appendix 1 Table 1. Summary of gross pathological findings in six Eurasian cranes (*Grus grus*) examined during the initial HPAIV H5N1 outbreak in Germany, autumn 2025*

Bird ID	Body condition	Pancreatic necrosis	Pulmonary edema	Epicardial hemorrhage	Proventricular hemorrhage	Other
#1	Good	x	-	x	x	Splenomegaly
#3	Good	x	x	-	-	Splenomegaly
#4	Good	x	x	x	-	Splenomegaly, renal congestion
#5	Good	x	x	-	-	Renal congestion
#6	Good	x	x	x	-	Renal congestion
#7	Good	x	-	-	-	Renal congestion

*Listed are body condition and the presence (x) or absence (–) of key gross lesions, including pancreatic necrosis, pulmonary edema, epicardial and proventricular hemorrhages, as well as additional findings recorded during necropsy

Appendix 1 Table 2. Semiquantitative scoring of influenza A viral antigen distribution, necrosis, and inflammation in multiple organs of three Eurasian cranes (*Grus grus*; #1/#3/#4) naturally infected with HPAIV H5N1*

Organ	Antigen score	Necrosis score	Inflammation score
Brain	3/4/4	2/2/2	2/2/2
Pancreas	3/3/3	3/3/3	2/2/2
Heart	2/2/2	0/0/0	0/0/0
Spleen	2/2/2	2/2/2	2/1/1
Liver	1/0/0	0/0/0	0/0/0
Kidney	1/1/2	1/1/2	1/1/2
Lung	1/1/1	0/0/0	0/0/0
Proventriculus	1/1/1	0/0/1	0/0/0
Gizzard	0/1/1	0/1/1	0/1/1
Small intestine	1/1/1	N/A	N/A
Large intestine	1/1/1	N/A	N/A

*Scores are provided for each organ as antigen score / necrosis score / inflammation score for cranes #1, #3, and #4, respectively. Antigen scores were assigned based on the extent of immunohistochemical (IHC) labeling: 0 = no antigen; 1 = focal/oligofocal (<5%, 1–3 foci; minimal); 2 = multifocal (6%–40%, >3 foci; mild); 3 = coalescing (41%–80%; moderate); 4 = diffuse (>80%; severe). Necrosis and inflammation were scored on consecutive HE sections using the same scale: 0 = no lesion; 1 = rare (<5%, 1–3 foci; minimal); 2 = multifocal (6%–40%, >3 foci; mild); 3 = coalescing (41%–80%; moderate); 4 = diffuse (>80%; severe). N/A indicates that the respective tissue was autolytic and could not be reliably assessed.
Note: Similar antigen and necrosis scores in some organs reflect the presence of necrotic foci within antigen-positive regions; however, necrosis did not involve all antigen-positive areas.

Appendix 1 Table 3. Cycle threshold results from different gene fragments of HPAIV H5 from 7 Eurasian cranes, Germany*

Bird	OPH	CL	Brain	Lung
Matrix gene				
# 1†	29.06	29.93	17.1	24.09
# 2	29.61	-	16.3	-
# 3†	26.9	26.66	15.93	27.16
# 4†	28.39	28.88	15.85	24.69
# 5	30.72	31.56	22.86	26.3
# 6	26.18	28.43	16.81	24.28
# 7	28.7	31.37	20.32	27.14
Hemagglutinin subtype				
# 1†	25.29	26.62	15.1	21.52
# 2	25.61	-	13.03	-
# 3†	23.71	24.18	13.19	25.16
# 4†	25.09	26.27	12.01	22.57
# 5	27.82	29.16	20.72	23.95
# 6	22.58	25.83	13.27	22.07
# 7	25.48	29.46	17.65	24
Neuraminidase subtype N1				
# 1†	25.67	26.49	14.84	20.66
# 2	26.18	-	12.6	-
# 3†	23.52	23.62	12.37	23.9
# 4†	25.02	25.43	11.12	22.02
# 5	27.43	28.12	20.09	23.63
# 6	23.08	25.86	12.1	21.2
# 7	24.93	28.39	16.59	23.71
Multibasic cleavage site of clade 2.3.4.4b HPAIV				
# 1†	26.05	27.45	15.25	21.58
# 2	26.17	-	13.29	-
# 3†	24.05	24.3	13.26	25.3
# 4†	26	26.93	12.12	22.78
# 5	27.99	29.31	21.08	24.24
# 6	23.02	26.19	13.82	22.03
# 7	26.83	29.72	17.69	24.6

*Postmortem investigation results from 7 Eurasian cranes (*Grus grus*; #1-#7) at the Friedrich-Loeffler-Institut. Brain, lung, cloacal swab, and oropharyngeal swab specimens were analyzed via RT-qPCR. The color coding, light to dark, represents increasingly high viral load; lower Ct values indicate higher viral loads. For individual #2 no cloacal swab and no lung sample could be taken due to feeding/scavenging damage at the carcass. CL, cloacal swab; OPH, Ct, cycle threshold; HPAIV, highly pathogenic avian influenza virus; OPH, oropharyngeal swab.
†Carcasses that had been applied to comprehensive histopathological investigations.

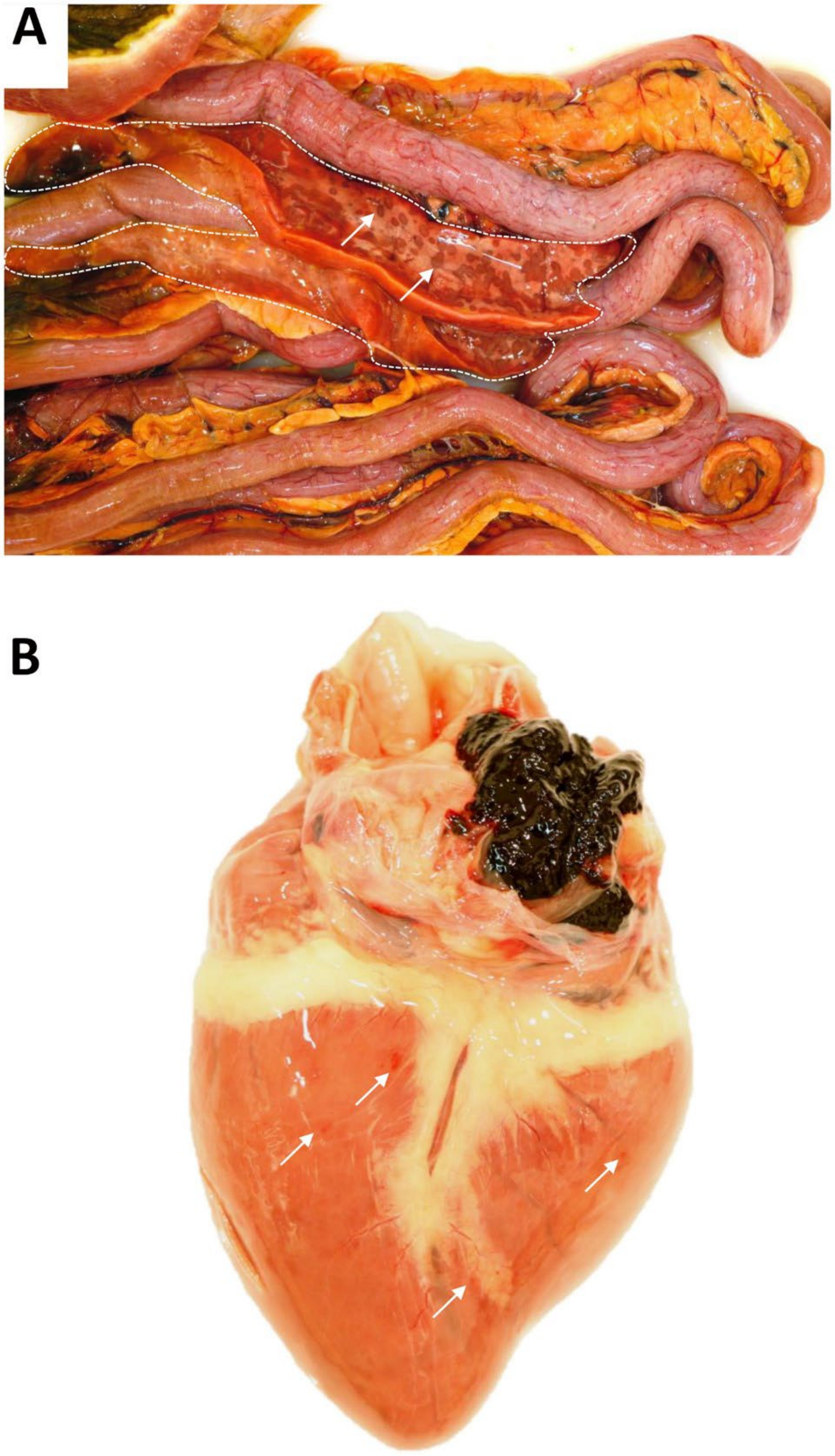

Appendix 1 Figure 1. Representative gross lesions in HPAIV H5N1–infected Eurasian cranes. (A) Pancreas (dashed outline) showing extensive multifocal to coalescing necrosis (arrows). (B) Multifocal epicardial hemorrhages (arrows).

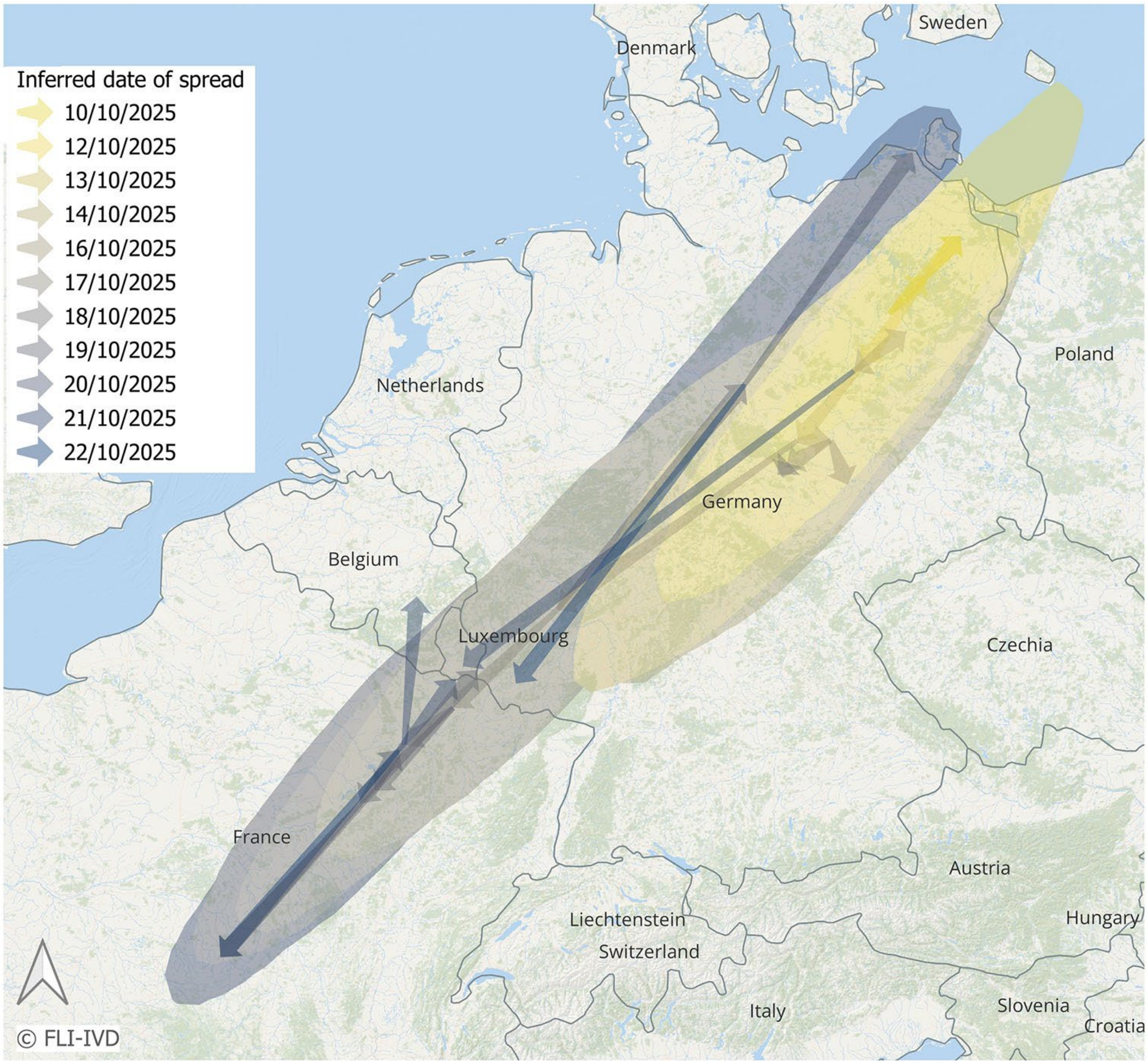

Appendix 1 Figure 2. Spread of H5N1 HPAI in Eurasian cranes inferred by spatial-time phylogeography with H5 HPAI viral genomes collected from European countries October 2025. Arrows indicated directed spread. Polygons show areas with 95% high posterior densities. Arrows and polygons are colored according to time.
